# Cysteine-Rich Angiogenic Inducer 61 Serves as a Potential Serum Biomarker for the Remission of Adult-Onset Still's Disease

**DOI:** 10.3389/fmed.2019.00266

**Published:** 2019-11-20

**Authors:** Yutong Su, Zhihong Wang, Junna Ye, Tienan Feng, Fan Wang, Huihui Chi, Zhuochao Zhou, Qiongyi Hu, Honglei Liu, Xiaobing Cheng, Hui Shi, Jialin Teng, Chengde Yang, Yue Sun

**Affiliations:** ^1^Department of Rheumatology and Immunology, Ruijin Hospital, Shanghai Jiao Tong University School of Medicine, Shanghai, China; ^2^Clinical Research Institute, Shanghai Jiao Tong University School of Medicine, Shanghai, China

**Keywords:** adult-onset Still's disease, cysteine-rich angiogenic inducer 61, disease activity, biomarker, disease remission

## Abstract

**Objective:** Adult-onset Still's disease (AOSD) is a rare, polygenic, systemic autoinflammatory disease. The aim of this study is to evaluate the serum levels of cysteine-rich angiogenic inducer 61 (Cyr61), a secreted, extracellular protein in AOSD patients.

**Methods:** A total of 60 AOSD patients (39 of active phase and 21 of inactive phase), 16 rheumatoid arthritis patients as a disease control, and 34 sex- and age-matched healthy control subjects (HC) were enrolled in the study. The data of the clinical manifestations and laboratory examinations were collected. The serum levels of Cyr61, interleukin (IL)-17, and IL-37 were detected by ELISA assay, and the serum levels of IL-10, IL-1β, IL-6, IL-18, and tumor necrosis factor (TNF)-α were examined by electrochemiluminescence assay.

**Results:** The serum levels of Cyr61 were significantly increased in inactive AOSD than those in active patients and HCs, and the levels of Cyr61 were dramatically increased after treatment. The levels of Cyr61 were inversely correlated with systemic score, the counts of leukocyte and neutrophil, and the levels of inflammatory cytokines (IL-1β, IL-6, and IL-17). Moreover, the levels of Cyr61 were higher in patients without the clinical symptoms of fever, skin rash, sore throat, arthralgia, and lymphadenopathy compared with those in patients with these symptoms.

**Conclusion:** The serum levels of Cyr61 were inversely correlated with disease activity in AOSD patients; thus, we proposed that Cyr61 was a biomarker for the remission of AOSD.

## Introduction

Adult-onset Still's disease (AOSD) is a rare, systemic, polygenic, autoinflammatory disorder characterized by the cardinal manifestations of fever, arthralgia or arthritis, skin rash, and increased counts of leukocytes and neutrophils (1–3). Patients may have serious complications such as macrophage activation syndrome (MAS), pulmonary arterial hypertension, and disseminated intravascular coagulopathy (1, 2). As the clinical manifestations of AOSD are consistent with several key common features of autoinflammatory diseases, it has been considered as the archetype of non-familial, polygenic, systemic autoinflammatory diseases in recent years (1).

Danger signals such as pathogen-associated molecular patterns (PAMPs) or damage-associated molecular patterns (DAMPs) are transmitted to macrophages and neutrophils that activate specific inflammasomes, leading to the production of IL-1β and IL-18. Furthermore, this process leads to intense innate immune cell activation and overproduction of several proinflammatory cytokines, including IL-6, IL-8, IL-17, and tumor necrosis factor (TNF)-α. This cytokine storm is the hallmark of AOSD, which plays a crucial role in the pathogenesis of AOSD (4). Meanwhile, we also demonstrated that the levels of anti-inflammatory cytokines IL-10 and IL-37 were increased in the serum of AOSD patients and positively correlated with disease activity (5, 6). Moreover, emerging evidences revealed that a series of mediators were considered as activity markers for AOSD, such as S100A8/9, S100A12, macrophage migration inhibitory factor (MIF), and HMGB1 (7–10). In addition, our previous study showed that microRNAs were potential biomarkers to distinguish AOSD from sepsis (11).

Cysteine-rich angiogenic inducer 61 (Cyr61), also termed cellular communication network factor 1 (CCN1), is a secreted extracellular protein mainly produced by stromal cells (12, 13). Multiple functions have been well-established on Cyr61 with regard to embryonic development, tumorigenesis, fibrosis, angiogenesis, cell proliferation, and migration (13, 14). However, the role of Cyr61 during inflammation is complicated. Our previous work reported that the expression of Cyr61 was increased in the synovium of rheumatoid arthritis (RA) patients and epidermis of psoriasis patients, and Cyr61 contributed to the cross-talk between immune cells and stromal cells during the development of autoimmune and inflammatory diseases (15–17). Cyr61 increases inflammatory cytokines IL-1β, IL-6, IL-8, and TNF-α expression in fibroblasts, keratinocytes, and macrophages *via* integrin receptors and downstream mitogen-activated protein kinase (MAPK) and nuclear factor (NF)-κB signals (16, 18, 19). Interestingly, Cyr61 also promotes tissue repair, a process accompanied by inflammation resolution (20). Cyr61 induced senescence of fibroblasts *via* upregulating p53 expression, which contributed to cutaneous tissue repair (21). Moreover, Cyr61 improved neutrophil efferocytosis by serving as a bridge between macrophages and neutrophils during wound healing (22). Recent clinical research suggested that Cyr61 served as a biomarker for RA and was negatively correlated with disease activity (23). Thus, the function of Cyr61 is controversial and undetermined in autoimmune and autoinflammatory diseases. Whether Cyr61 contributes to the pathogenesis of AOSD is still unclear. Here, we explored the levels of Cyr61 in the serum of AOSD patients and discussed the relationship between Cyr61 and clinical and laboratory features of the disease.

## Materials and Methods

### Patients

A total of 60 AOSD patients were retrospectively enrolled in this study. The patients were consecutive AOSD patients who visited the Department of Rheumatology and Immunology, Ruijin Hospital, Shanghai Jiao Tong University School of Medicine from September 2015 to March 2018. The diagnosis of AOSD was according to the criteria of Yamaguchi et al. (24) after malignancies, infections, and other autoimmune diseases were excluded. Thirty-four age- and sex-matched volunteers were enrolled as healthy control subjects (HC). The diagnosis of RA was according to the 2010 American College of Rheumatology (ACR) classification criteria (23). The study was performed in accordance with the Declaration of Helsinki and the principles of good clinical practice. Biological samples were obtained under a protocol approved by the Institutional Research Ethics Committee of Ruijin Hospital (identifier 2016-62), Shanghai, China. Informed consent was obtained from the recruited subjects.

Medical histories including clinical and laboratory characteristics were collected from all subjects. Each sample underwent a complete blood count, erythrocyte sedimentation rate (ESR), C-reactive protein (CRP), rheumatoid factor, antinuclear antibody, ferritin, and liver function tests. Follow-up samples were collected from 11 patients with active AOSD after the resolution of disease activity. AOSD disease activity was assessed according to the systemic disease score method (25), which comprised 12 disease manifestations, as follows: fever, evanescent rash, sore throat, arthritis, myalgia, pleuritis, pericarditis, pneumonitis, lymphadenopathy, hepatomegaly or abnormal liver function tests, elevated leukocyte count >15,000/μl, and serum ferritin >3,000 μg/l. Patients with AOSD were considered to have clinically active stage if they had a fever and/or an inflammatory arthralgia/arthritis and/or any suggestive cutaneous lesions and/or a sore throat. Their AOSD was otherwise considered inactive (26).

### Cytokine Measurement

Serum levels of Cyr61 were measured by sandwich ELISA assay using an R&D Quantikine ELISA Kit (R&D Systems, Inc., Minneapolis, Canada) according to the manufacturer's instructions. Serum levels of IL-1β, IL-6, TNF-α, and IL-18 were measured using an electrochemiluminescence assay (Meso Scale Discovery, MSD, Rockville, USA) according to the previous reports (6).

### Statistical Analysis

The results were analyzed by GraphPrism 7.00 software (GraphPad Software Inc., San Diego, USA). The Shapiro-Wilk normality test (*n* ≤ 50) or KS normality test (*n* > 50) was used to analyze whether the data fit for the parametric contribution. The data were expressed as mean ± SD for parametric data or median with interquartile range (IQR) for non-parametric data. The serum levels of Cyr61 before and after treatment from individual patients were analyzed by paired *t*-test. The associations between the serum levels of Cyr61 and different variables were analyzed by the non-parametric Spearman correlation test. The non-parametric Mann-Whitney *U* test was used to compare differences between each group. The analyses were carried out under the two-sided principle. The differences were considered significant when *P* < 0.05.

## Results

### Increased Serum Levels of Cyr61 in Inactive AOSD Patients

**Figure 1:**
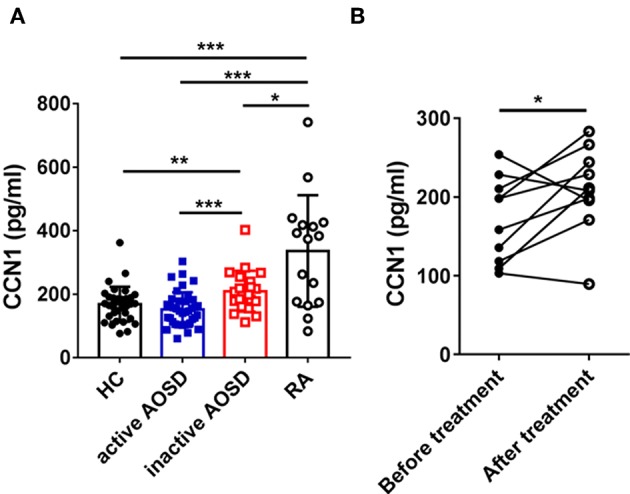
The serum levels of cysteine-rich angiogenic inducer 61 (Cyr61) in adult-onset Still's disease (AOSD) patients are shown. **(A)** The serum levels of Cyr61 in healthy control subjects (HC) (•, *n* = 34) and active AOSD (■, *n* = 39), inactive AOSD (□, *n* = 21), and rheumatoid arthritis (RA) (°, *n* = 16) patients are shown. The non-parametric Mann–Whitney *U*-test was used to compare differences between each group, and data represent median with interquartile range (IQR). **(B)** The serum levels of Cyr61 in patients before (•) and after (°) clinical treatment (*n* = 11) were analyzed by paired *t*-test. *P < 0.05, **P < 0.01, ***P < 0.001.

To examine the serum levels of Cyr61 in AOSD, a total of 60 AOSD patients including 39 active patients and 21 inactive patients, 16 RA patients, and 34 HCs were enrolled in the current study. The clinical and laboratory information of these specimens was listed in Table 1. Using the ELISA assay, we found that the serum levels of Cyr61 were not altered in total AOSD patients (171.8 ± 63.8 pg/ml, *n* = 60) compared to those of HC (168.2 ± 54.9 pg/ml, n = 34, *p* > 0.05) (Supplementary Figure 1). Further analysis showed that significantly increased levels of Cyr61 were detected in the serum of inactive AOSD patients compared with those of active AOSD patients and HC (Figure 1A). Besides, the serum levels of Cyr61 were higher in RA patients compared with those in AOSD patients and HC (Figure 1A). Moreover, the levels of Cyr61 were dramatically increased in inactive patients after treatment during follow-up (Figure 1B). Next, we compared the levels of Cyr61 in 39 active AOSD patients with or without treatment at the time of enrollment to evaluate the effect of treatment on the levels of Cyr61. The results showed that treatment did not influence the levels of Cyr61 in active patients (Supplementary Figure 2), so the change of Cyr61 after remission was mainly due to the improvement of the disease activity in AOSD patients.

**Table 1:** Clinical characteristics of patients at the time of enrollment.

	**AOSD (*****n*** **= 60)**		**RA (*n* = 16)**	**HC** **(*n* = 34)**
	**Active** **(*n* = 39)**	**Inactive** **(*n* = 21)**		
Age (year)	36.0 ± 12.5	36.0 ± 13.8	55.5 ± 12.4	39.0 ± 10.8
Gender (F/M)	29/10	15/6	13/3	24/10
Duration (months)	15.0 ± 22.2	17.0 ± 23.4	18.1 ± 16.4	
**Clinical features**				
Fever	35 (89.7)	0 (0.0)		
Sore throat	27 (69.2)	0 (0.0)		
Skin rash	31 (79.5)	0 (0.0)		
Lymphadenopathy	28 (71.8)	3 (14.3)		
Splenomegaly	15 (38.5)	0 (0.0)		
Hepatomegaly	1 (2.6)	0 (0.0)		
Pericarditis	8 (20.5)	0 (0.0)		
Pleuritis	10 (25.6)	0 (0.0)		
Pneumonia	18 (46.2)	0 (0.0)		
Myalgia	14 (35.9)	0 (0.0)		
Arthralgia	35 (89.7)	0 (0.0)	16 (100.0)	
Arthritis	7 (17.9)	0 (0.0)	16 (100.0)	
Systemic score	5.9 ± 1.9	0.2 ± 0.4		
**Laboratory markers**				
Hemoglobin, g/L	110.7 ± 26.3	131.8 ± 17.2	122.9 ± 12.8	
Leukocyte, × 10^9^/L	16.7 ± 5.7	8.0 ± 2.7	6.8 ± 2.2	
Platelet, × 10^9^/L	279.2 ± 115.0	232.4 ± 76.4	221.9 ± 68.0	
ESR, mm/h	68.6 ± 26.8	19.6 ± 29.6	47.2 ± 28.8	
CRP, mg/L	93.1 ± 60.0	23.6 ± 24.5	14.7 ± 36.1	
ALT, U/L	61.5 ± 60.4	23.0 ± 11.6	14.7 ± 5.9	
AST, U/L	46.9 ± 25.6	18.6 ± 7.4	21.0 ± 2.6	
Ferritin, ng/ml	2779.0 ± 4286.0	210.2 ± 207.4		
ANA positivity	6 (14.6)	1 (4.8)	5 (31.2)	
RF positivity	2 (4.9)	0 (0.0)	14 (87.5)	
ACPA positivity	0 (0.0)	1 (4.8)	9 (56.3)	
**Treatments**				
Steroids and sDMARDs naïve	18 (46.2)	8 (38.1)	3 (18.8)	
Low dosage of steroid monotherapy	2 (5.1)	1 (4.8)	0 (0.0)	
High dosage of steroid monotherapy	4 (10.3)	0 (0.0)	0 (0.0)	
sDMARD(s)	0 (0.0)	3 (14.2)	8 (50.0)	
Combination therapy, steroids + sDMARD(s)	15 (38.4)	9 (42.9)	5 (31.2)	

*All values are presented as n (percent) or mean ± SD*.

*AOSD, adult onset Still's disease; HC, healthy control; ESR, erythrocyte sedimentation rate; CRP, C-reactive protein; AST, aspartate transaminase; ALT, alanine transaminase; ANA, anti-nuclear antibody; RF, rheumatoid factor; ACPA, anticitrullinated peptide antibodies; sDMARD, synthetic disease-modifying antirheumatic drug*.

### The Serum Levels of Cyr61 Were Inversely Correlated With Disease Activity in AOSD Patients

**Figure 2:**
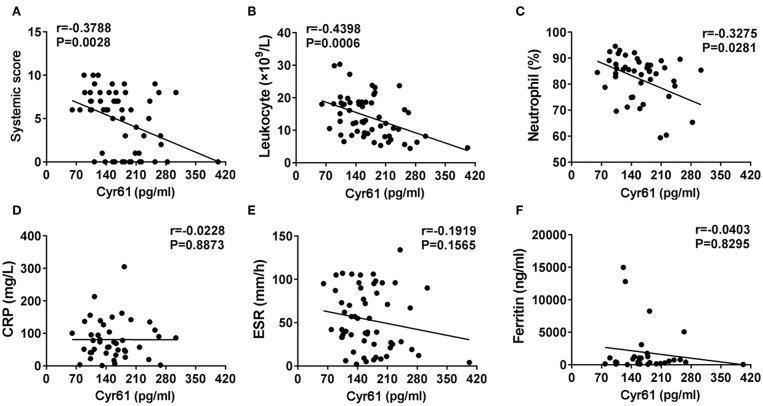
The correlations between the serum levels of cysteine-rich angiogenic inducer 61 (Cyr61) and disease activity in adult-onset Still's disease (AOSD) patients are shown. The correlations between serum levels of Cyr61 and systemic score **(A)**, the counts of leukocytes **(B)** and neutrophils **(C)**, and C-reactive protein (CRP) **(D)**, erythrocyte sedimentation rate (ESR) **(E)**, and ferritin levels **(F)** were analyzed by the non-parametric Spearman correlation test.

To explore the correlation between serum levels of Cyr61 and disease activity in AOSD, we analyzed the relationship of the serum levels of Cyr61 and the systemic score. The levels of Cyr61 were inversely correlated with the systemic score (*r* = −0.3788; *p* = 0.0028) (Figure 2A). Meanwhile, we found that the serum levels of Cyr61 were inversely correlated with the counts of leukocyte (*r* = −0.4398; *p* = 0.0006) and neutrophil (*r* = −0.3275; *p* = 0.0281) but were not significantly correlated with CRP (*r* = −0.0228; *p* = 0.8873), ESR (*r* = −0.1919; *p* = 0.1565), and ferritin (*r* = −0.0403; *p* = 0.8295) (Figures 2B–F).

### The Levels of Cyr61 Were Higher in Patients Without the Cardinal Disease Symptoms in AOSD Patients

**Figure 3:**
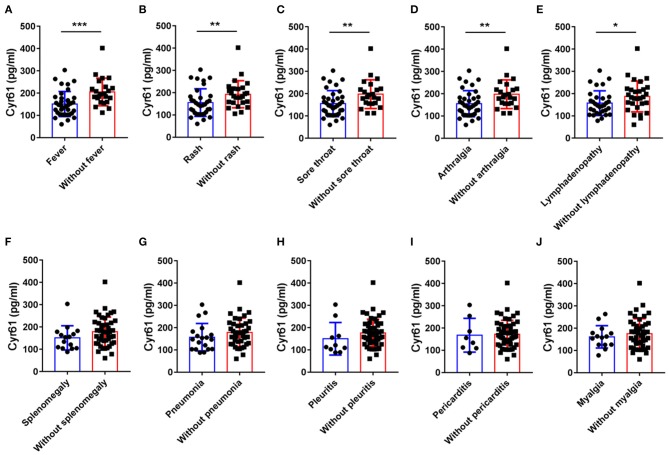
The serum levels of cysteine-rich angiogenic inducer 61 (Cyr61) according to disease manifestations in adult-onset Still's disease (AOSD) patients are shown. The serum levels of Cyr61 in AOSD patients with (•) or without (■) the symptoms of fever **(A)**, skin rash **(B)**, sore throat **(C)**, arthralgia **(D)**, lymphadenopathy **(E)**, splenomegaly **(F)**, pneumonia **(G)**, pleuritis **(H)**, pericarditis **(I)**, and myalgia **(J)** are shown. The non-parametric Mann–Whitney *U*-test was used to compare differences between each group, and data represent median with interquartile range (IQR). *P < 0.05, **P < 0.01, ***P < 0.001.

To assess associations between serum levels of Cyr61 and clinical manifestations in patients with AOSD, serum levels of Cyr61 were compared among patients with and those without certain clinical features. As shown in Figure 3, the levels of Cyr61 were significantly higher in AOSD patients who did not have fever, skin rash, sore throat, arthralgia, and lymphadenopathy compared with patients who had these clinical manifestations (*p* < 0.05); however, there was no significant difference of serum levels of Cyr61 between patients with or without splenomegaly, pneumonia, pleuritis, pericarditis, and myalgia. Together, these data demonstrated that serum levels of Cyr61 correlated significantly with the remission of cardinal clinical features in AOSD patients.

### The Relationships of Cyr61 With Inflammatory Cytokines in AOSD Patients

**Figure 4:**
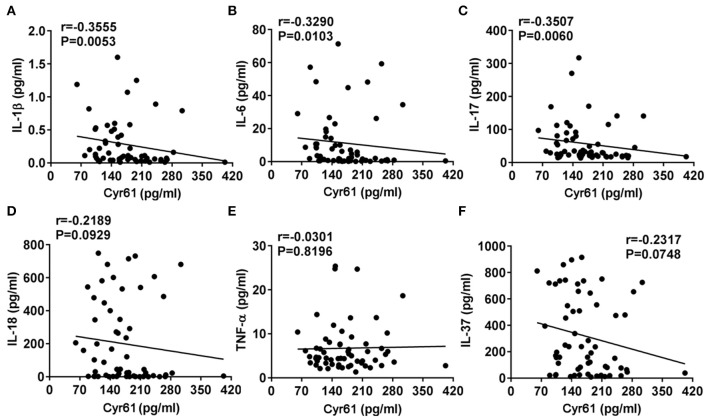
The correlations between the serum levels of Cyr61 and inflammatory cytokines in adult-onset Still's disease (AOSD) patients are shown. The associations between the serum levels of Cyr61 and interleukin (IL)-1β **(A)**, IL-6 **(B)**, IL-17 **(C)**, IL-18 **(D)**, tumor necrosis factor (TNF)-α **(E)**, and IL-37 **(F)** were analyzed by the non-parametric Spearman correlation test.

It has been well-demonstrated that the levels of proinflammatory cytokines (IL-1β, IL-6, IL-18, and TNF-α) were increased in AOSD and contributed to the pathogenesis of AOSD (1). Thus, we analyzed the correlation between the levels of Cyr61 and inflammatory cytokines. As shown in Figure 4, the serum levels of Cyr61 exhibited a strong inverse correlation with the serum levels of IL-1β (*r* = −0.3555, *p* = 0.0053), IL-6 (*r* = −0.3290, *p* = 0.0103), IL-17 (*r* = −0.3507, *p* = 0.0060), whereas there was no correlation with the levels of IL-18 (*r* = −0.2189, *p* = 0.0929), TNF-α (*r* = −0.0301, *p* = 0.8196), and IL-37 (*r* = −0.2317, *p* = 0.0748).

### Fever and Leukocytes Were Independently Associated With the Levels of Cyr61

Furthermore, we used univariate and multivariate linear regression analyses to evaluate the correlations between serum Cyr61 levels and clinical features, laboratory values, and inflammatory cytokines. Multiple linear regression analysis showed that fever (β coefficient = −74.416, 95% CI: −141.063, −7.770; *p* = 0.029) and leukocytes (β coefficient = −3.416, 95% CI: −6.588, −0.244; *p* = 0.035) were independently associated with Cyr61 (Table 2).

**Table 2:** Linear regression models to explore a specific clinical or laboratory item associated with Cyr61.

**Analytical data**	**β Coefficient**	**95% CI**	***p***
**Univariate analysis**			
Fever	−60.423	(−90.163, −30.684)	0.000
Arthralgia	−52.679	(−83.418, −21.939)	0.001
Arthritis	4.558	(−47.204, 56.320)	0.861
Skin rash	−43.682	(−75.057, −12.306)	0.007
Sore throat	−52.814	(−83.204, −22.425)	0.001
Lymphadenopathy	−30.018	(−62.330, 2.294)	0.068
Splenomegaly	−27.595	(−65.289, 10.099)	0.148
Hepatomegaly	−68.172	(−196.765, 60.420)	0.293
Pericarditis	−4.630	(−53.510, 44.251)	0.850
Pleuritis	−26.179	(−70.244, 17.887)	0.239
Pneumonia	−21.499	(−56.780, 13.783)	0.227
Myalgia	−13.684	(−52.818, 25.449)	0.487
Systemic score	−7.627	(−12.781, −2.472)	0.004
Leukocyte, × 10^9^/L	−64.322	(−98.934, −29.709)	0.001
ESR, mm/h	−0.303	(−0.771, 0.165)	0.200
CRP, mg/L	−0.005	(−0.291, 0.280)	0.971
Ferritin, ng/ml	−0.003	(−0.009, 0.004)	0.414
IL−10, pg/ml	3.882	(−0.881, 8.645)	0.108
IL−1β, pg/ml	−22.028	(−64.310, 20.255)	0.301
IL−6, pg/ml	−0.402	(−1.381, 0.578)	0.415
TNF–a, pg/ml	0.252	(−2.815, 3.318)	0.870
IL−18, ng/ml	−0.027	(−0.095, 0.040)	0.424
**Multivariate analysis**			
Fever	−4.416	(−141.063, −7.770)	0.029
Arthralgia	4.340	(−50.373, 59.054)	0.874
Skin rash	−17.042	(−67.220, 33.136)	0.498
Sore throat	−29.668	(−68.442, 9.106)	0.131
Systemic score	12.140	(−0.158, 24.438)	0.053
Leukocyte, × 10^9^/L	−3.416	(−6.588, −0.244)	0.035

*CRP, C-reactive protein; ESR, erythrocyte sedimentation rate*.

## Discussion

Cyr61 is a secreted, matricellular protein, which is the first member of Cyr61-CTGF-NOV protein family (13). Cyr61 contributes to multiple physiological and pathological processes, such as embryonic development, angiogenesis, cell proliferation and migration, tumorigenesis, wound healing, and inflammation, by binging to diverse integrin receptors and activating different downstream signaling pathways (12, 13). In recent years, elevated levels of Cyr61 had been found in many autoimmune and inflammatory diseases, such as RA, systemic lupus erythematosus (SLE), inflammatory bowel disease (IBD), Sjogren's syndrome (SS), and psoriasis (15, 17, 27–29). However, the profiles of Cyr61 in AOSD, a multigenic, systemic autoinflammatory disease, are still unknown. Here, we investigated the serum level of Cyr61 and its association with disease activity in AOSD patients, we proposed that Cyr61 was a potential biomarker of AOSD disease activity.

In the present study, we found that the serum levels of Cyr61 were significantly increased in inactive AOSD patients compared with those in active patients. Moreover, during serial follow-up, serum levels of Cyr61 were elevated in the inactive phase after effective treatment. This inverse correlation was also confirmed in AOSD patients classified by different clinical manifestations. The serum levels of Cyr61 were increased in AOSD patients who had disease remission after fever, skin rash, sore throat, arthralgia, and lymphadenopathy improved. The serum levels of Cyr61 were inversely correlated with systemic score and proinflammatory cytokines, such as IL-1β, IL-6, and IL-17. Taken together, we concluded that the serological Cyr61 level was inversely correlated with AOSD disease activity. Coincidentally, a recent clinical cohort study of RA showed that the levels of Cyr61 were dramatically lower in patients with higher disease activity, and it showed a significant increase at 12 weeks in ACR responders (ACR20/50/70) compared with those in non-responders (23). Similarly, a previous report evaluated the potential value of Cyr61 in SLE-associated pulmonary arterial hypertension patients. It revealed that patients with higher levels of Cyr61 had better survival than those with a lower Cyr61 level. Besides, we noticed that the levels of Cyr61 showed no correlation with the levels of CRP, ESR, and ferritin maybe because Cyr61 is a stromal cell-derived factor, not an inflammatory cytokine in the initial stage of inflammation in AOSD.

The negative correlation of Cyr61 with disease activity is complicated and partially resulted from the diverse functions of Cyr61. It is well-known that Cyr61 triggered inflammation by upregulating the expression of inflammatory cytokines, such as TNF-α, IL-1β, and IL-6 in both immune cells and nonimmune cells *via* different integrin receptors and MAPK/NF-κB signal pathway (16, 18, 19). However, multiple studies revealed the function of Cyr61 on the limitation of inflammation and tissue repair. In liver inflammation, Cyr61 facilitated the expansion of HLA-DR^−/low^CD33^+^CD11b^+^ myeloid-derived suppressor cells (MDSCs) *via* α_m_β_2_/STAT3 signal and Cyr61/GM-CSF-induced MDSCs inhibited T-cell proliferation significantly (30). In murine experimental autoimmune myocarditis, overexpression of Cyr61 decreased the cardiac disease score and inhibited macrophage and lymphocyte migration without changing the chemokine and cytokine expression (31). As well, in dextran sodium sulfate (DSS)-induced colitis, misfunction of Cyr61 resulted in high mortality and Cyr61 treatment promoted mucosal healing by accelerating intestinal epithelial cell proliferation (27). During the biliary injury, Cyr61 promoted the proliferation and differentiation of cholangiocytes through αvβ5/NF-κB/JAG1 pathway to improve tissue repair (32). In addition, Cyr61 bound both integrins α_v_β_3_ and α_v_β_5_ on macrophages and phosphatidylserine on neutrophils to promote neutrophil efferocytosis (22) and induced fibroblast senescence by activating p53 and ROS pathway and inhibited fibrosis in wound healing (21). Whether Cyr61 plays a protective role in the pathogenesis of AOSD needs further study. As Cyr61 was increased in inactive AOSD patients, we speculated that Cyr61 participated in the development of AOSD, especially involved in the process of inflammation resolution and tissue repair during AOSD remission.

To our knowledge, the inflammatory stimuli, such as TNF-α, IL-1β, IL-6, and IL-17, which were increased in the serum of AOSD patients, promoted Cyr61 expression (15, 18, 33); however, non-parametric Spearman correlation analysis revealed that the serum levels of Cyr61 were inversely correlated with the levels of IL-1β, IL-6, and IL-17 (Figure 4). The reason why Cyr61 levels were not increased in inflammatory circumstances might be due to the tight control of Cyr61 expression. MicroRNA is one of the key posttranscriptional controllers in regulating gene expression, and several microRNAs were reported to target Cyr61, such as miR-22, miR-181c, miR-155, and miR-142-5p (34–37). In our previous work, the expression of miR-142-5p was dramatically increased in plasma from active AOSD than that from inactive AOSD (11). As Cyr61 is the direct target of miR-142-5p, it might partially contribute to the lower expression of Cyr61 in active patients of AOSD with systemic inflammation.

It is important to acknowledge that there are some limitations in our study. First, the power of the study is limited by a relative small sample size and the retrospective design, as a result, prospective studies with a larger sample size are needed in the future. Second, active AOSD patients enrolled were not all treatment naive, and not every active patient was followed up, which may reduce the power of statistical analysis. Finally, the mechanism of Cyr61 in the pathogenesis of AOSD is still unknown; further experimental study is still needed.

In conclusion, our study first reported the increased levels of Cyr61, a matricellular protein in the serum of inactive AOSD patients, we found that its expression was inversely correlated with disease activity. As Cyr61 is secreted by stroma cells, such as fibroblast cells, keratinocytes, and endothelial cells (15, 17, 38), the injured tissues after inflammation destruction might provide the local source of Cyr61 in remission phase, contributing to tissue repair. However, given the fact that Cyr61 is expressed in distinct cell types and different times, the proinflammatory and anti-inflammatory characters were exhibited diversely, the pathogenesis involved needs to be studied thoroughly. Thus, we propose that Cyr61 might be a potential biomarker for the remission of AOSD.

## Data Availability Statement

The datasets generated for this study are available on request to the corresponding author.

## Ethics Statement

The studies involving human participants were reviewed and approved by The Institutional Research Ethics Committee of Ruijin Hospital (identifier 2016-62), Shanghai, China. The patients/participants provided their written informed consent to participate in this study.

## Author Contributions

YSun and YSu conceived of the study and participated in its design and coordination. ZW and JY carried out the ELISA and performed the statistical analysis. HC, ZZ, QH, HL, XC, HS, and JT collected samples and contributed to data acquisition, analysis, and critical review for intellectual content. TF made the statistics for all data. YSun, YSu, and CY drafted the manuscript and revised the manuscript. All authors read, revised, and approved the final manuscript.

### Conflict of Interest

The authors declare that the research was conducted in the absence of any commercial or financial relationships that could be construed as a potential conflict of interest.
