# Supplementary material for: Cysteine-Rich Angiogenic Inducer 61 Serves as a Potential Serum Biomarker for the Remission of Adult-Onset Still's Disease
Source: Front Med (Lausanne). 2019 Nov 20;6:266. doi: 10.3389/fmed.2019.00266 (PMC6879423; doi:10.3389/fmed.2019.00266)
Supplement: Supplementary file 1 [file Data_Sheet_1.DOCX]

Supplementary Material

## Supplementary Figures


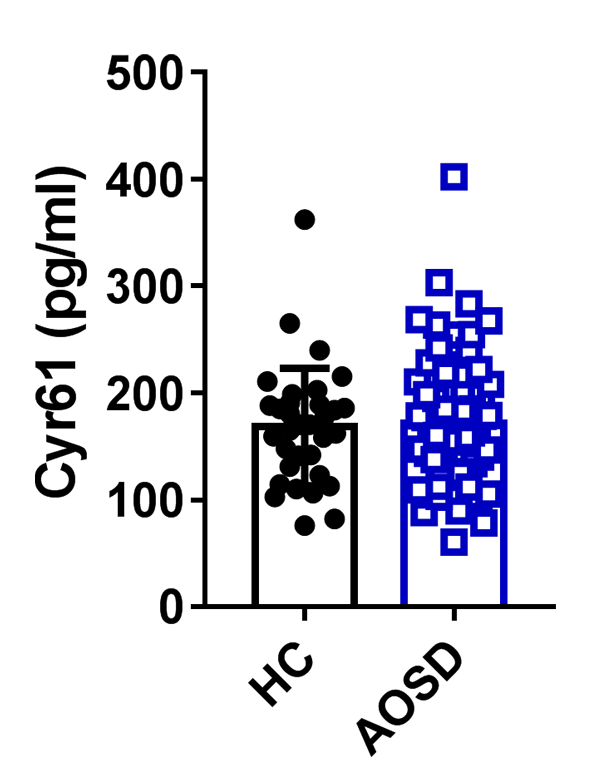


**Supplementary Figure 1.** The serum levels of Cyr61 in total AOSD patients. The serum levels of Cyr61 in HC (●, *n* = 34) and AOSD (□, *n* = 60) patients. Date represent median with interquartile range (IQR).

.
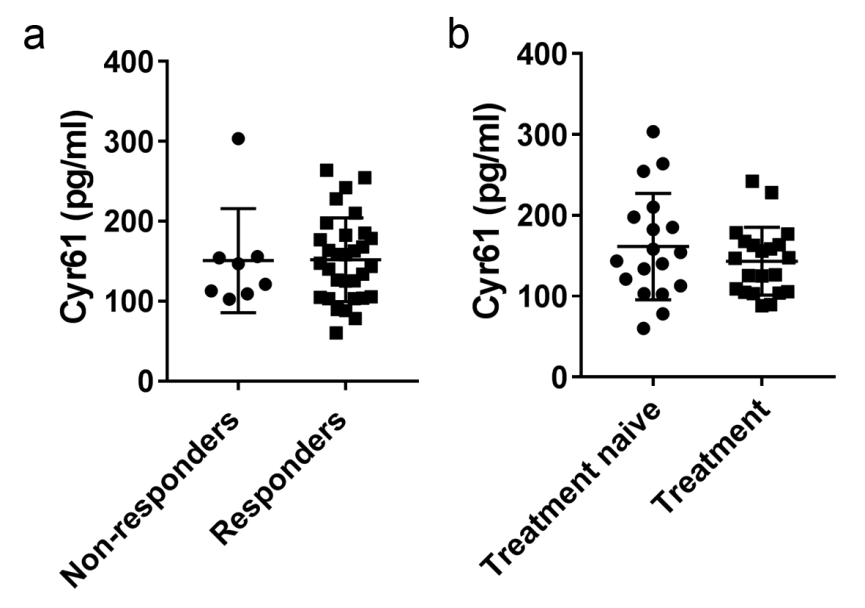


**Supplementary Figure 2.** The levels of Cyr61 in treatment naive patients (●, *n* = 18) or patients treated with [glucocorticoid](javascript:;) or DMARDs or combined therapy (■, *n* = 21).
